# Exposure Estimation for Risk Assessment of the Phthalate Incident in Taiwan

**DOI:** 10.1371/journal.pone.0151070

**Published:** 2016-03-09

**Authors:** Chu-Chih Chen, Shu-Li Wang, Ming-Tsang Wu, Yin-Han Wang, Po-Chin Huang, Bai-Hsiun Chen, Chien-Wen Sun, Chi-Kung Ho, Yang-Chih Shih, Ming-Neng Shiu, Wen-Harn Pan, Mei-Lien Chen, Ching-Chang Lee, Chao A. Hsiung

**Affiliations:** 1 Division of Biostatistics and Bioinformatics, Institute of Population Health Sciences, National Health Research Institutes, Miaoli, Taiwan; 2 National Institute of Environmental Health Sciences, National Health Research Institutes, Miaoli, Taiwan; 3 Department of Public Health, College of Health Sciences, Kaohsiung Medical University, Kaohsiung, Taiwan; 4 Department of Family Medicine, Kaohsiung Medical University Hospital, Kaohsiung, Taiwan; 5 Center of Environmental and Occupational Medicine, Kaohsiung Municipal Hsiao-Kang Hospital, Kaohsiung, Taiwan; 6 Department of Laboratory Medicine and Pediatrics, Kaohsiung Medical University Hospital, Kaohsiung Medical University, Kaohsiung, Taiwan; 7 Graduate Institute of Clinical Medicine, College of Medicine, Kaohsiung Medical University, Kaohsiung, Taiwan; 8 Department of Public Health, Kaohsiung Medical University, Kaohsiung, Taiwan; 9 Department of Occupational and Environmental Medicine, Kaohsiung Medical University Hospital, Kaohsiung, Taiwan; 10 Department of Health, Kaohsiung City Government, Kaohsiung, Taiwan; 11 Ministry of Health and Welfare, Taipei, Taiwan; 12 Division of Preventive Medicine and Health Services Research, Institute of Population Health Sciences, National Health Research Institutes, Miaoli, Taiwan; 13 Institute of Biomedical Sciences, Academia Sinica, Taipei, Taiwan; 14 Institute of Environmental and Occupational Health Sciences, College of Medicine, National Yang Ming University, Taipei, Taiwan; 15 Department of Environmental and Occupational Health, College of Medicine, National Cheng Kung University, Tainan, Taiwan; 16 Research Center of Environmental Trace Toxic Substance, National Cheng Kung University, Tainan, Taiwan; Institute for Health & the Environment, UNITED STATES

## Abstract

**Background:**

In May 2011, di(2-ethylhexyl) phthalates (DEHP) and, to a lesser extent, di-iso-nonyl phthalate (DiNP) were found to have been illegally used for many years in Taiwan as clouding agents in foods including sports drinks, juice beverages, tea drinks, fruit jam/nectar/jelly, and health or nutrient supplements.

**Objective:**

To estimate the DEHP exposure for the study participants for the follow-up epidemiological study and health risk assessment.

**Methods:**

A total of 347 individuals possibly highly exposed to phthalate-tainted foods participated in the study. Exposure assessment was performed based on the participants' responses to a structured questionnaire, self-report of exposure history, urinary metabolite concentrations, and DEHP concentration information in 2449 food records. A Bayesian statistical approach using Markov chain Monte Carlo simulation was employed to deal with the uncertainties in the DEHP concentrations of the contaminated foods and the participants' likelihood of being exposed.

**Results:**

An estimated 37% and 15% of children younger than 12 years old were exposed to DEHP at medium (20–50 *μg* / *kg*_*bw* / *day*) and high AvDIs (50–100 *μg* / *kg*_*bw* / *day*), respectively, prior to the episode (9% and 3% in adults, respectively). Moreover, 11% of children and 1% of adults were highly exposed (> 100 *μg* / *kg*_*bw* / *day*), with a maximum of 414.1 *μg* / *kg*_*bw* / *day* and 126.4 *μg* / *kg*_*bw* / *day*, respectively.

**Conclusions:**

The phthalate exposure-associated adverse health effects for these participants warrant further investigation. The estimation procedure may be applied to other exposure assessment with various sources of uncertainties.

## Introduction

The phthalate family of chemicals is widely used in plastic production, primarily as plasticizers [1,2], and as components of personal-care products such as lotions and cosmetics, and pharmaceuticals and medical devices [3,4,5]. Exposure to phthalates increases the risk of allergies and asthma [6,7], has an adverse impact on children's neurodevelopment, anogenital distance, and thyroid function [6,8,9], and may adversely affect levels of reproductive hormones [10]. Among the phthalates, di(2-ethylhexyl) phthalate (DEHP) is the most common plasticizer and accounts for 40% of soft polyvinyl chloride (PVC) [11]. Regarding regulatory limits for exposure to DEHP, the tolerable daily intake (TDI) according to the European Food Safety Authority is 50 *μg* / *kg*_*bw* / *day* [12], and the reference dose (RfD) recommended by the US Environmental Protection Agency is 20 *μg* / *kg*_*bw* / *day*.

On April 7 2011, during a routine examination, a laboratory staff member of the Taiwan Food and Drug Administration (TFDA), Department of Health (DOH, now the Ministry of Health and Welfare) accidentally detected a DEHP (not included in the formal check list) level of approximately 600 ppm in a probiotic supplement [13]. Further investigation later found that DEHP and/or di-isononyl phthalate (DiNP) had been illegally used by two upstream large perfumery-chemical companies for more than a decade as a cheaper substitute for emulsifiers or clouding agents, which were then distributed to major food manufacturers and used in various food products in Taiwan [2,13,14]. To protect consumers from further exposure, the DOH ordered that food products must be removed from stores immediately if a DEHP/DiNP concentration >1 ppm was detected, and the updated information was posted on the TFDA website. For public information, TFDA classified the contaminated food products into five categories: (1) sport drinks; (2) tea drinks; (3) juice beverages; (4) fruit jam, nectar, or jelly; and (5) health or nutrition supplements in the form of capsules, tablets, or a powder. The DOH further announced that starting on May 31, 2011, all food products confirmed to be contaminated with plasticizers must be removed from the shelves and recalled. To address the general public's concerns about possible health effects, the DOH established clinics in 128 hospitals across the country to provide consultations and basic physical examinations. Individuals were transferred to specialty clinics if they were suspected of being highly exposed or had abnormal test results. To investigate the associated health effects, a multi-year study project was funded by the DOH, and public health experts were invited to conduct the Risk Assessment of the Phthalate Incident in Taiwan (RAPIT). Because of the limited number of DiNP-tainted foods detected, the magnitude of the detected concentrations, and the relatively high TDI of DiNP (150 *μg* / *kg*_*bw* / *day*), we focused on the estimation of average daily intake (AvDI) of DEHP exposure. The reconstructed AvDI is intended to be used, together with the participants' biomarker measurements (S1 File), for the follow-up epidemiological studies and quantitative health risk assessments.

Because DEHP metabolizes to mono-(2-ethylhexyl) phthalate (MEHP) and other secondary metabolites very rapidly, with a half-life less than 24–48 h [15], it is technically not possible to assess the participants' prior DEHP exposure more than one year ago based on their current urine metabolite measurements, as is the case in most phthalate studies [7,11,15,16]. Therefore, in addition to urine DEHP metabolite measurements for background exposure, this study relied on information from various sources, including food frequency questionnaires (FFQ), self-reported exposure histories, and detected concentrations in the tainted foods to reconstruct the AvDI of DEHP. The approach inevitably involved various sources and degrees of uncertainties, including the true DEHP concentrations in the tainted foods, participants' responses to the FFQ for having been exposed to certain tainted foods, and exposure duration, amount, and frequency, etc, especially after a time lag of more than one year. A Bayesian statistical approach using Markov chain Monte Carlo (MCMC) simulation by setting separate prior probability distributions was employed to deal with these uncertainties.

## Materials and Methods

### Study subjects

The study subjects who were suspected of being highly exposed or had abnormal physical examination results were recruited by the RAPIT project from the specialty clinics of the 3 participating hospitals (the DOH Hospitals in Taipei and Taichung and the Kaohsiung Medical University Hospital) after the incident. Additionally, those who complained to the Consumers Foundation and claimed to be victims of the tainted foods were encouraged to participate in the study. In total, there were 347 participants, including 237 children (<12 years old), 13 adolescents (12 to 18 years old), and 97 adults (≥18 years old) who underwent an exposure assessment questionnaire interview, blood and urine collection and a physical examination during a clinical visit between August 2012 and February 2013. Consumption information for children under age 12 years old were given by their parents or caregivers. All participants gave their written informed consent after receiving written and oral information about the study. Written informed consent on behalf of the participated children was obtained from their parent or caregiver. The study was approved by the Research Ethics Committee of the National Health Research Institutes of Taiwan.

### Data

#### Phthalate concentration data

**Fig 1:**
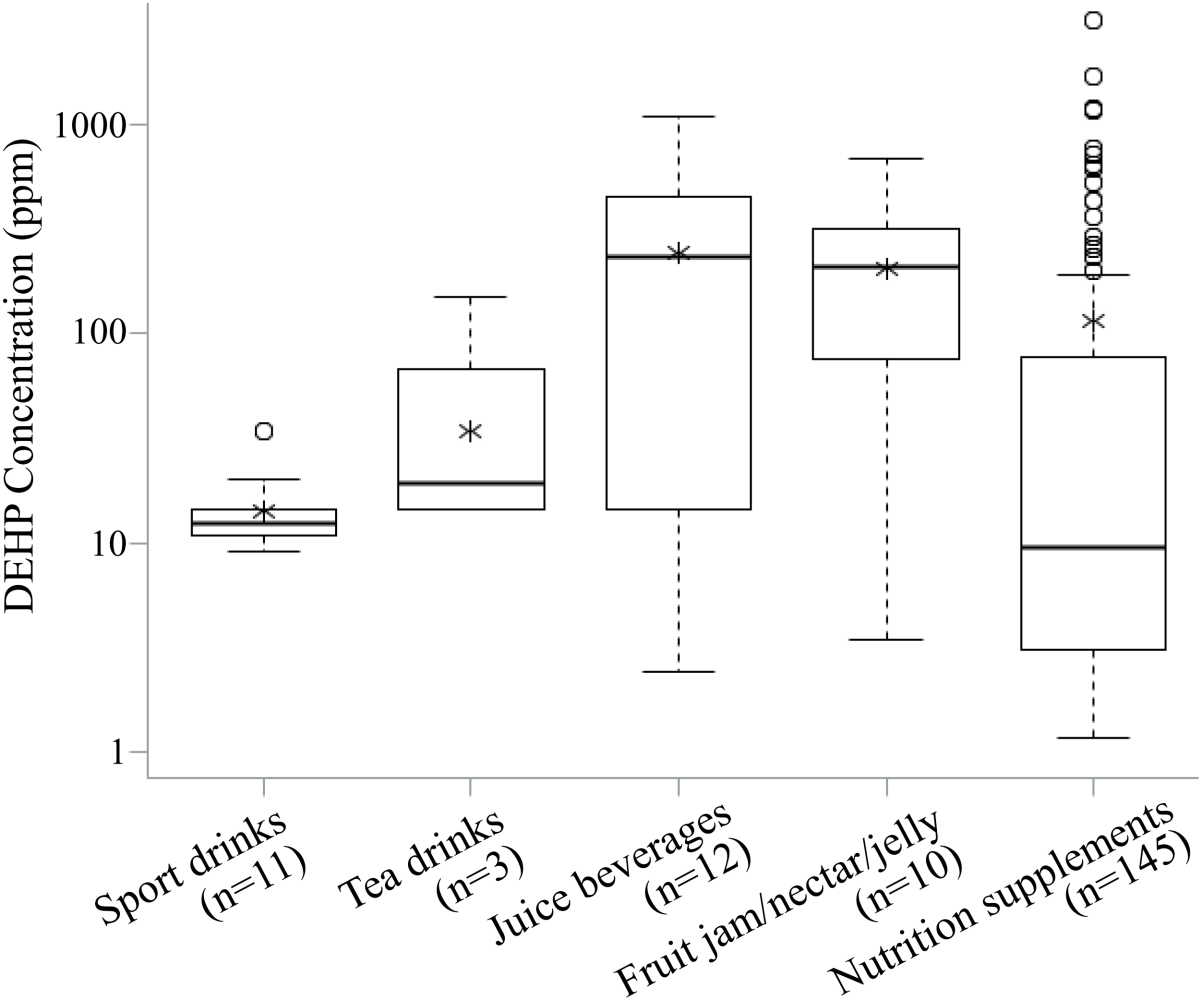
Box plots of the DEHP concentrations for the five contaminated food categories. The upper and lower bars are the 1.5 inter-quartile range and the minimum DEHP concentration, respectively. The "*" is the mean concentration. Each circle represents one record of DEHP concentration in the contaminated food products. The unit of the vertical axis is on the log-scale.

Phthalate concentrations for 522 and 1927 food product records examined prior to May 31, 2011, from the TFDA and the Bureau of Health of Kaohsiung City (KBOH), respectively, were obtained. Of these, 114 and 39 food products examined by the TFDA and KBOH, respectively, were contaminated with DEHP. Multiple measurements of the same food product were merged into a single record for that product. For about half of the tainted food products, only partial information of concentration > 1 ppm was available due to quick screening at the time. Depending on the food category, the detected DEHP concentrations ranged from 1.2 ppm to over 3000 ppm. Among the tainted food products from various manufacturers or different production dates, concentrations varied by more than tenfold between the minimum and maximum. Fig 1 shows the box plots of the detected DEHP concentrations in the five food categories. The mean DEHP concentrations ranged from 14 ppm in the sport drinks to 224.5 ppm in the juice beverages, which were several orders in magnitude higher than those found in the U.S. and European countries [17–19].

#### Exposure assessment questionnaire

To assess the dietary exposure history of the study participants, a structured FFQ, consisting of one question for each of the five food categories (Q1-Q5), was designed. Based on the exposure amount, frequency, duration, and degree of uncertainty, each question was divided into several subcategories: whether the participant had been exposed (yes, 10 points; not sure, 1 point; no, 0 points), consumption frequency (2 times or less per week, 1 point; 3 to 5 times, 3 points; 6 times or more, 5 points), and consumption duration (less than half a year, 1 point; between half a year and 1 year, 2 points; between 1 and 2 years, 3 points; over 2 years, 4 points). The corresponding scores from each subcategory were multiplied. A total of 1000 points were possible with a maximum of 200 points from each of the five food categories. In addition, the participants were asked to provide a history of exposure to the tainted foods with as much detail as possible, including the name of the product(s), producer(s), consumption frequency, quantity consumed per exposure, and duration of exposure. A list of the tainted food products officially announced was shown to the participants for confirmation during the interview.

#### Urine sample for background exposure to DEHP

Spot urine samples from the participants were collected at the clinic. Three measured DEHP metabolites (mono 2-ethylhexyl phthalate (MEHP), mono 2-ethyl-5-hydroxyhexyl phthalate (MEHHP), and mono 2-ethyl-5-oxohexyl phthalate (MEOHP)) in the urine samples were analyzed to assess the participant's current background DEHP exposure status [20, 21]. Briefly, a urine sample (100 μl) was thawed, sonicated for 10–15 min, and was loaded into a glass vial (2 ml) that contained ammonium acetate (20 μl, Sigma Aldrich Laboratories, Inc., St. Louis, MO, USA), β-glucuronidase (10 μl, Escherichia coli-K12, Roche Biomedical, Mannheim, Germany) and a mixture of isotopic (^13^C_4_) phthalate metabolite standards (100 μl) which were purchased from Cambridge Isotope Laboratories, Inc. (Andover, MA, USA). An on-line system was used that was coupled with liquid chromatography/ electrospray ionization tandem mass spectrometry (LC—ESI-MS/MS) (Agilent 1200/ API4000, Applied Biosystems, Foster City, CA, USA). Two columns were used in our on-line system: one C18 column (Inertsil ODS-3, 33*4.6 mm, 5 μm, GL Science, Tokyo, Japan) for extraction and clean-up of the collected samples and one analytical column (Inertsil Ph, 150 *4.6 mm, 5 μm, GL Science, Tokyo, Japan) for separation of different phthalate metabolites. A negative, multiple reaction monitoring model was used for mass detection. The ion pairs of each phthalate metabolite are listed as follows: MEHP (277/134), MEHHP (293/121), MEOHP (291/143), with detection limits of 0.7, 0.3, and 0.3 ng mL^-1^, respectively. One blank, repeat and quality control (QC) sample were included in each batch of samples analyzed. Concentration of blank sample shall be below 2 fold of method detection limit. The QC sample was spiked in pooled urine sample with a mixture of phthalate metabolite standards (20–50 ng/ml) in each sample. The relative percent difference of repeat sample and recovery of QC sample shall be below ±30%.

The daily intake of DEHP was estimated using the sum of the creatinine-adjusted concentrations of the three metabolites, which were converted to moles per gram before summing. The calculation of AvDI for adolescents and children was based on the following equation:

$$AvDI_{ENV}\left(\mu g/kg\_bw/day\right)=\frac{UE_{sum}\left(\mu mol/g\right)\times CE\left(g/day\right)}{F_{UE}\times BW\left(kg\right)}\times MW_{DEHP}$$ (1)

where UE_sum_ (urinary excretion) is the sums of molar urinary excretion of MEHP, MEHHP, and MEOHP in micromoles per gram creatinine, CE is the gender-specific body height-based reference values for urinary creatinine excretion in children and adolescents aged 3–18 years old (molecular weight transformed) [22], F_UE_ (= 0.32 for MEHP, MEHHP, and MEOHP) is the molar fraction of excreted metabolite relative to total intake at 24-h post-dosing [23], BW is the participant's body weight, and MW_DEHP_ is the molecular weight of DEHP [15]. The AvDI for adults was calculated based on the daily creatinine excretion rate CE (23 *mg/kg/day* and 18 *mg/kg/day* body-weight normalized for men and women, respectively) [15], i.e.,

$$AvDI_{ENV}\left(\mu g/kg\_bw/day\right)=\frac{UE_{sum}\left(\mu mol/g\right)\times CE\left(mg/kg/day\right)}{F_{UE}\times 1000(mg/g)}\times MW_{DEHP}$$ (2)

#### Estimation of total AvDI for the study participants

The exposure assessment questionnaire only provided information on whether participants were exposed, approximate exposure frequency, and possible exposure duration. In contrast, the self-report gave more specific detailed information on the product name, producer, consumption amount, frequency, and explicit duration (especially for the nutrition supplement). Therefore, we estimated AvDIs of DEHP separately from the questionnaire (*AvDI*_*QN*_) and self-report (*AvDI*_*SF*_) (if it was provided by a participant or child's caregiver). Assume that the lifestyle of the participants remained approximately the same after the episode and that the tainted foods had been effectively removed, background exposure *AvDI*_*ENV*_ was estimated by converting the urine DEHP metabolite measurements using Eqs (1)–(2). A total daily exposure of DEHP for the participants was calculated by summing the estimates from all the three different sources of information as follows:

$$AvDI_{ALL}=AvDI_{QN}+AvDI_{SF}+AvDI_{ENV}$$ (3)

The AvDI of DEHP based on the participant's questionnaire responses and self-report was estimated using the equation

$$AvDI=\frac{\sum_{i=1}^{5}Y_{i}\times M_{i}\times EF_{i}\times ED_{i}}{AT\times BW_{b}}$$ (4)

where *Y*_*i*_, *M*_*i*_, *EF*_i_, and *ED*_*i*_ are the concentration (mg/L or ppm), consumption amount (ml or g), exposure frequency (times per day), and exposure duration (day), respectively, of the i-th food product category, *i* = 1,…,5; *AT* is the average time of exposure in days, and *BW*_*b*_ is the participant's body weight in kg on May 31, 2011, the official last day of the incident announced by the DOH.

For each of the five food categories, an AvDI (*μg* / *kg*_*bw* / *day*) was calculated from the questionnaire outcomes. A reconstructed *AvDI*_*QN*_ was then obtained by summing the AvDIs for each of the food categories. To avoid double counting and to lower uncertainty, the *AvDI*_*QN*_ of a specific food category was counted as 0 if a self-report of exposure(s) to tainted foods in that category was available.

Uncertainties in the DEHP concentration *Y*_*i*_ of the tainted food and the participants likelihood of having been exposed were mainly estimated by using Bayesian models describe in the following section. The consumption amount *M*_*i*_ (ml) for the sport drinks, tea drinks and juice beverages was based on the standard volume for a bottled/cup drink. The consumption amount for fruit jams/nectar/jelly (g) was simulated from a normal distribution with a mean based on daily normal consumption amount. The consumption amount for nutrition supplements (g) was mainly ascertained from participants' self-reports of dosages consumed. For the participants who did not provide a self-report or specific consumption dosage, the amount was simulated from the average reported dosage (S1 File, Section 3). For self-report with unknown intake amounts and consumption frequencies, the corresponding quantities were estimated from the means reported by the other participants. For the participants without self-report, consumption frequencies and durations were approximated by taking the medians from the questionnaire outcomes (e.g., 9 months if the answer was between half a year and one year).

The average time of exposure *AT* (days) for AvDI_QN_ was determined by the maximum exposure duration of the five food categories from the questionnaire outcomes. For the participants with a self-report, *AT* was calculated from the sum of consumption durations of various food products if several tainted foods were consumed at discontinuous time periods (especially the health or nutrition supplements). If the participant consumed several tainted food products simultaneously, the *AT* was estimated from the maximum of the exposure durations.

Because the body weights of the studied children are expected to grow at the same rate as general population, we adjusted their body weight on May 31, 2011, by the median growth rate coefficient (GRC). That is, the body weight, *BW*_*b*_ (kg), was obtained by adjusting the participant's body weight measured during the clinic visit by a median GRC [24], *γ*_*j*_(*a*,*t*), where *j* = 0 or 1 if the participant is a girl or boy, respectively, *a* is participant's current age, and *t* is the time interval between the clinic visit and May 31, 2011. For example, if a boy, age 5, weighed 20 kg at the clinic visit, which was 1.5 years after May 31, 2011, then the corresponding GRC is *γ*_1_(5,1.5) = 18.3/15.3≅1.2 and *BW*_*b*_ = 20/1.2≅16.7 (kg), where 18.3 kg and 15.3 kg are the median weights of boys of 5 and 3.5 years of age in Taiwan, respectively (Table 3 of [24]). We assumed that the body weight of adult participants remained the same during the period, i.e., *γ*_*j*_ (*a*,*t*) = 1 if *a* ≥ 18 years old. To reflect the uncertainty in growth rate, the GRC was simulated from a normal distribution with a mean of the calculated GRC and a standard deviation defined by the coefficient variation of 0.16 times the mean for each child.

Because there was a time lag of more than one year for the clinic visit, an additional exposure index AvDI* with a window period was calculated by replacing the average time *AT* (day) with AT* (day), which includes an extra time lag for biomarker measurements (i.e., AT* = *AT* + time lag), and by replacing the body weight, *BW*_*b*_ (kg), of Eq (4) with the participant's present body weight, *BW* (kg).

#### Bayesian statistical models for uncertainties in DEHP concentrations and dietary intake exposures

**Fig 2:**
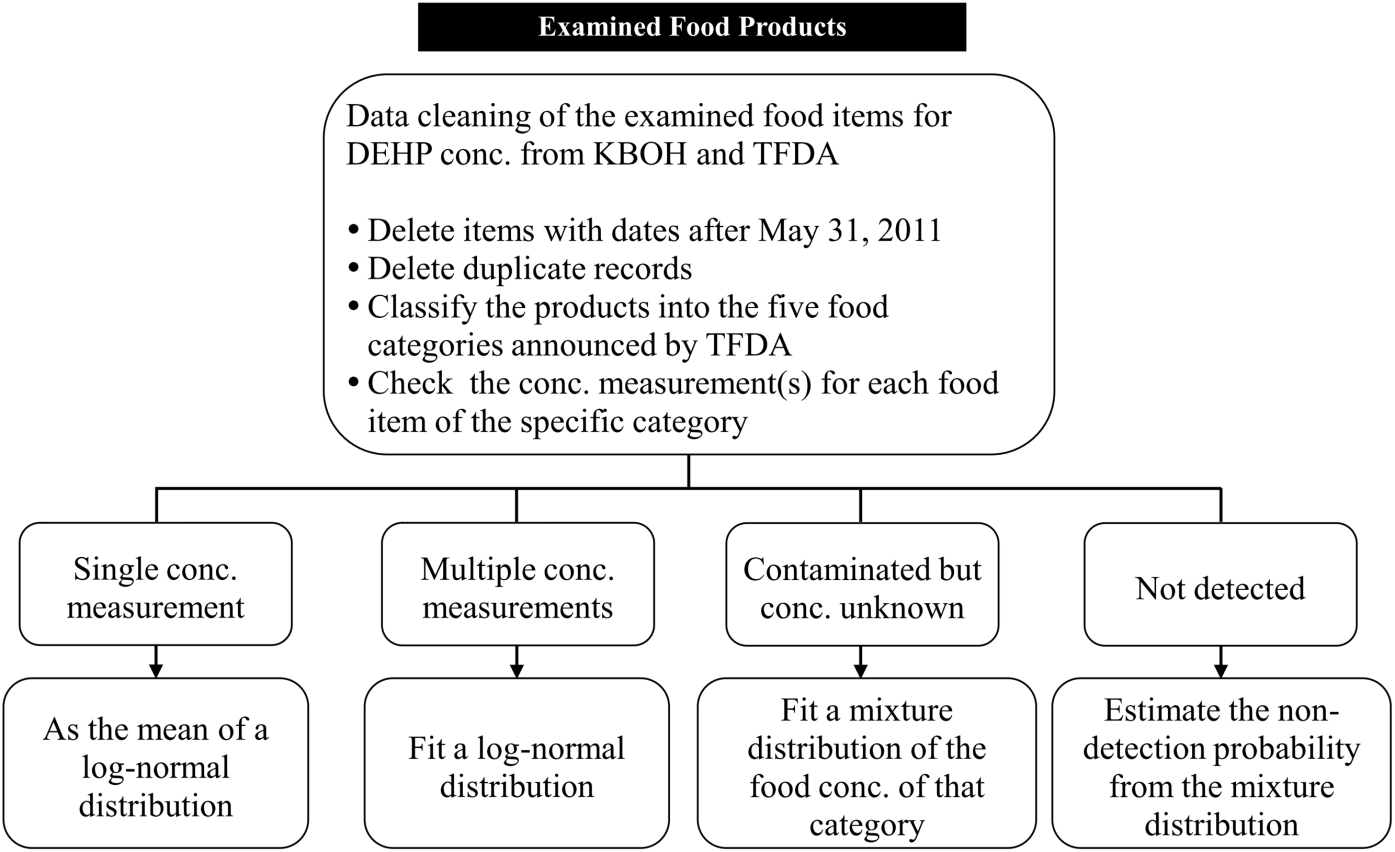
Data management and distribution estimate for the DEHP-tainted food concentration measurements from KBOH and TFDA.

A Bayesian statistical procedure using MCMC simulation was employed to deal with the complexity of uncertainties in the DEHP concentrations of the contaminated foods and exposure scenarios of the participants. Different prior lognormal distributions were used to describe the measured DEHP concentrations of the tainted foods, and a mixture distribution for average concentration was obtained by weighing the proportions of various product measurements for each food category. The details are given in S1 File, Section 4. Fig 2 shows the data management and estimation procedure for the DEHP concentration.

**Fig 3:**
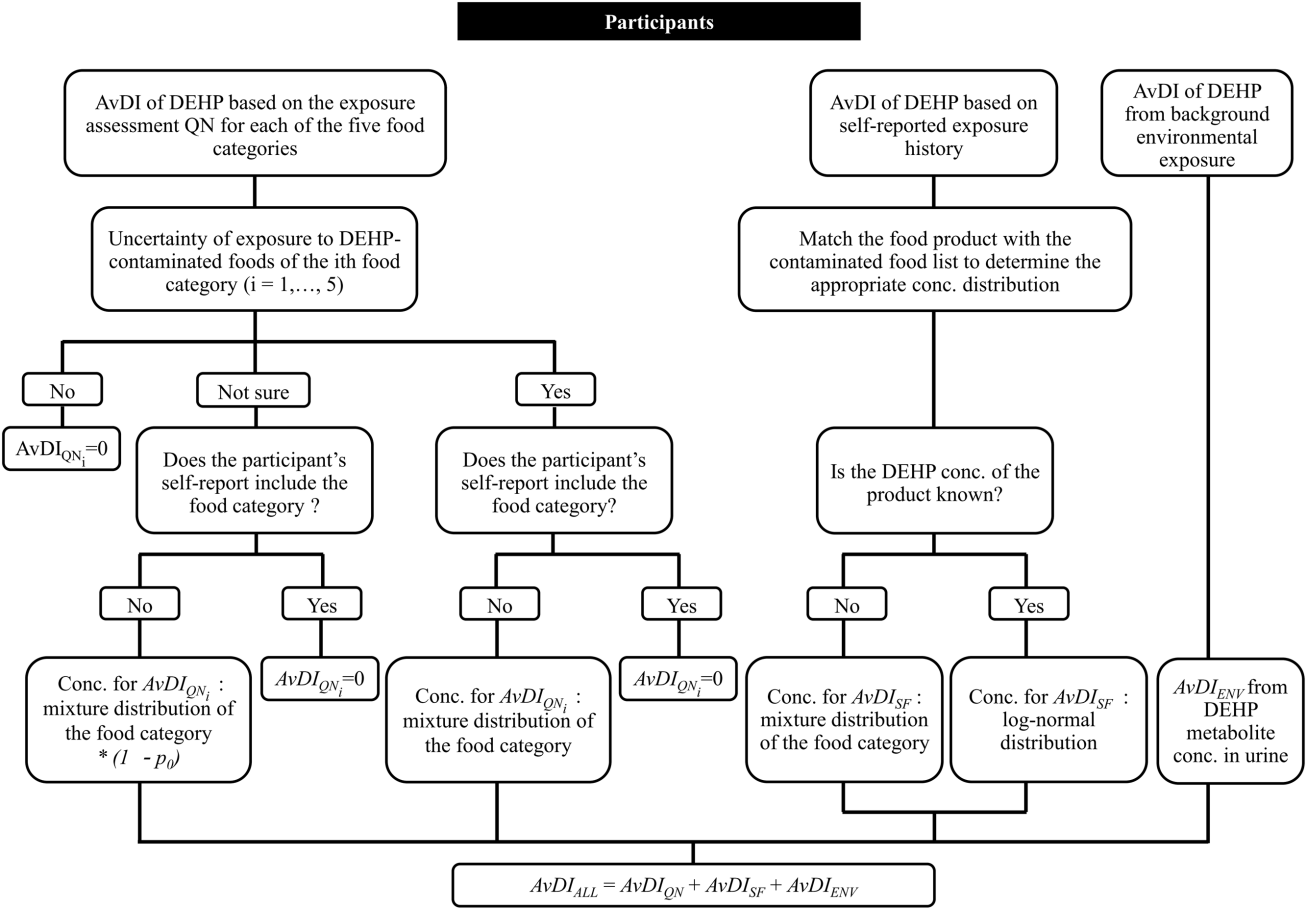
Flowchart of the classification and estimation procedure. The overall AvDI of DEHP was as ascertained from the exposure assessment questionnaire, self-reported exposure history, and metabolite concentrations in urine.

Fig 3 shows the classification of uncertainties of the participants based on their responses to the study questionnaire and self-report, as well as the reconstruction procedure.

## Results and Discussion

### DEHP concentrations in the contaminated food products

Using Bayesian MCMC simulations, we estimated the detection rate *p*_*D*_ (= 1 − *p*_*ND*_), the odds ratio *exp*(*β*), the mean DEHP concentration *E*(*Y*), the probabilities *p*_0_, *p*_1_, and the expected exposure concentration, *E*(*C*). The results are summarized in Table 1. Overall, the proportions of food samples being detected ranged from 0.014 (1/73) for juice beverages (KBOH) to 0.340 (16/47) for nutrition supplements (TFDA). The overall estimated detection rate *p*_*D*_ for the five food categories was less than 0.1, and the odds ratios ranged from 0.8 to 44.1. Among the non-detected food products, the probability that the DEHP concentration was between 0 ppm and 1 ppm was 0.014 to 0.038. In those participants who were unsure of their exposure, the estimated exposure ranged from 0.5 ppm for juice beverages to 6.6 ppm for health or nutrition supplements.

**Table 1:** Number of examined food samples provided by the KBOH and the TFDA and the parameter estimates by food category.

Food category	KBOH^b^		TFDA^b^		*p*_*D*_^b^	exp(*β*)^b^	*E*(*Y*)^b^	*p*_1_^b^	*p*_*0*_^b^	*E*(*C*)^b^
	Detected^c^	Total	Detected^c^	Total						
Sport drinks	0	7	4	22	0.058 (0.013–0.143)	44.1 (6.1–312.6)	12.9 (6.5–35.6)	0.014	0.941 (0.855–0.987)	0.8 (0.1–5.2)
Tea drinks	2	46	1	29	0.042 (0.013–0.089)	0.8 (0.1–5.7)	19.1 (1.4–89.1)	0.013	0.958 (0.910–0.987)	0.8 (0.0–8.0)
Juice beverages^a^	1	73	9	109	0.014 (0.013–0.015)	6.3 (0.9–44.1)	35.3 (1.6–204.5)	0.038	0.985 (0.984–0.986)	0.5 (0.0–3.3)
Fruit jam, nectar, or jelly	1	25	9	58	0.041 (0.037–0.044)	5.6 (0.8–40.9)	123.1 (3.3–785.0)	0.019	0.959 (0.955–0.962)	5.1 (0.1–35.5)
Health or nutrition supplements	110	1166	16	47	0.105 (0.088–0.122)	4.9 (0.7–34.7)	62.1 (29.5–159.8)	0.018	0.893 (0.875–0.910)	6.6 (2.7–19.9)

^a^The concentration estimate was divided by a constant ratio of 7 for concentrated juice.

^b^KBOH, Bureau of Health of Kaohsiung City; TFDA, Taiwan Food and Drug Administration; exp(β), odds ratio for the non-detection rate of TFDA over KBOH; *p*_*D*_, probability of DEHP concentration being≥1 ppm; *E(Y)*, expected DEHP exposure concentration of the tainted foods; *p*_*1*_, probability of DEHP concentration being between 0 and 1 ppm; *p*_*0*_, probability of DEHP concentration being 0; *E(C)*, expected DEHP exposure concentration for overall foods of the category.

^c^Contaminated food products with several DEHP concentration measurements were counted as the same detected food product.

We classified the detected DEHP concentrations into several disjoint intervals because of the broad range for each food category. For tainted food products with an unknown concentration, a mixture distribution was applied with weights proportional to the established lognormal distributions. This approach is appealing because the clouding agents made from DEHP were produced in batches, and the mean concentrations would likely vary with production dates. Moreover, the various food products from the manufacturers might also be obtained from different suppliers, each with their own formula. Therefore, the DEHP concentration in the tainted foods is a mixture distribution from various suppliers with different manufacturing dates.

### Estimated AvDIs of DEHP

**Fig 4:**
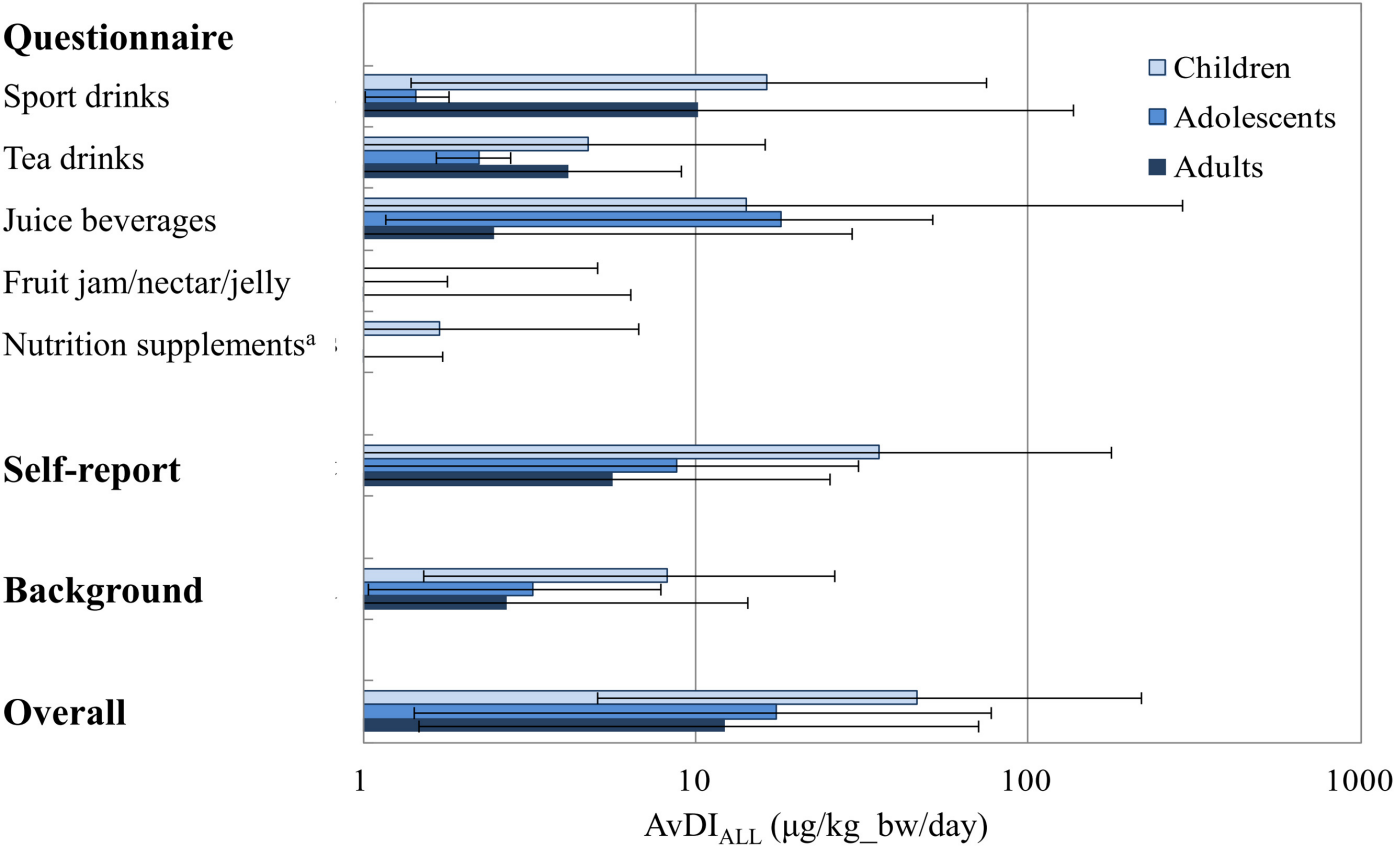
Mean estimated AvDIs of DEHP by each food category and by self-report, and background exposure. The unit of the vertical axis is on the log-scale. ^a^The main exposure from this food category was calculated as *AvDI*_*SF*_.

Of the participants, 237, 13, and 97 of the children, adolescents, and adults, respectively, completed the study questionnaire. Ten children and one adult with no urine sample, and one adolescent and one adult with negligible consumption frequency were excluded from analysis. A total of 227, 12, and 95 children, adolescents, and adults, respectively, were eligible for estimation of *AvDI*_*ALL*_. Using Eqs (1)–(4) and (8), the participants' AvDIs of DEHP from tainted-foods were reconstructed. Fig 4 shows the estimated mean AvDIs by age group for each of the five food categories from the questionnaire (*AvDI*_*QN*_), the self-report (*AvDI*_*SF*_), and the background exposure (*AvDI*_*ENV*_), as well as the overall intake *AvDI*_*ALL*_.

The mean *AvDI*_*SF*_ in children was exceptionally high because many of the children had a regular intake of tainted nutritional supplements. It is quite possible that these children had probiotic supplements following doctor's advice because of allergy-related health problem and thus were recruited for the study. Altered thyroid functions for the highly exposed children were observed in a previous study [9]. Clinical follows for potential developmental effects deserve more efforts. In comparison, the estimated *AvDI*_*QN*_ s of the tainted sport drinks and juice beverages were greater than 10 *μg* / *kg*_*bw* / *day*, and were relatively small or close to 0 for the other three categories. The estimated *AvDI*_*QN*_ s of the tainted fruit jams and nutritional supplements were close to 0 because of uncertainty of exposure. Except for tea drinks, children tended to have higher exposures than adults, including from background exposures (*AvDI*_*ENV*_). Although the estimated *AvDI*_*ENV*_ was relatively small compared to *AvDI*_*QN*_ and *AvDI*_*SF*_, its contribution to exposure cannot be ignored. Avoiding environmental exposures such as contacts of plastic toys and vinyl tiles for children also needs more attention.

The AvDIs were calculated with and without consideration of the window period because the associated health biomarker measurements might have been altered after the window period [9]. Table 2 classifies the estimated *AvDI*_*ALL*_ into low (< 20 *μg* / *kg*_*bw* / *day*), medium (20–50 *μg* / *kg*_*bw* / *day*), high (50–100 *μg* / *kg*_*bw* / *day*), and very high (>100 *μg* / *kg*_*bw* / *day*) according to the RfD suggested by the US EPA and the TDI by the EU. Among the children, 85, 83, and 34 (37%, 37%, and 15%, respectively) had AvDIs in the low, medium, and high exposure groups, respectively. In contrast, 82 (86%) of the adults had AvDIs in the low exposure group. Only 4% had AvDIs exceeded 50 *μg* / *kg*_*bw* / *day*. Noticeably, 11% and 1% of children and adults, respectively, had an AvDI > 100 *μg* / *kg*_*bw* / *day*, and the maximum AvDIs among children and adults were as high as 414.1 *μg* / *kg*_*bw* / *day* and 126.4 *μg* / *kg*_*bw* / *day*, respectively (S4 Table). A chi-square test showed that the difference between children and adults was highly significant (p-value< 0.0001). Because of the small sample size of adolescents, the reconstructed AvDIs of this age group are not discussed.

**Table 2:** Classification of the estimated overall AvDIs (with and without adjusting the window period) of DEHP into low (< 20 *μg* / *kg*_*bw* / *day*), medium (20–50 *μg* / *kg*_*bw* / *day*), high (50–100 *μg* / *kg*_*bw* / *day*), and very high exposure (>100 *μg* / *kg*_*bw* / *day*) groups.

		Exposure group				
		Low	Medium	High	Very High	
		(< 20)	(20–50)	(50–100)	(>100)	Total
Without window period	Children	85 (37%)	83 (37%)	34 (15%)	25 (11%)	227
	Adolescents	9 (75%)	2 (17%)	1 (8%)	0 (0%)	12
	Adults	82 (86%)	9 (9%)	3 (3%)	1 (1%)	95
With window period	Children	143 (63%)	63 (28%)	15 (7%)	6 (3%)	227
	Adolescents	11 (92%)	1 (8%)	0 (0%)	0 (0%)	12
	Adults	89 (94%)	6 (6%)	0 (0%)	0 (0%)	95

After considering the window period, 63 (28%), 15 (7%), and 6 (3%) of the children were still in the medium, high, and very high exposure groups, respectively. Most of the adults (94%) had AvDIs below 20 *μg* / *kg*_*bw* / *day*. The results showed that there were still close to 40% of the children exposed to phthalates at medium to very high level even after adjusting for the lagged window period after the episode. Because of the known adverse health effects [6–9], long-term follow-up studies on the participated children is warranted.

The background exposure AvDI_ENV_ estimated from participant's urinary DEHP metabolite concentrations was based on the assumption that the tainted food products had been removed from marketplace, and the level should reflect background food intake exposures and lifestyle exposures such as cosmetic uses. The correlation coefficients (p-values) between *AvDI*_*ENV*_ and *AvDI*_*QN*_ and *AvDI*_*SF*_ were 0.09 (0.32) and 0.10 (0.14), respectively, in children; –0.32 (0.48) and 0.43 (0.16), respectively, in adolescents; and 0.14 (0.27) and 0.08 (0.46), respectively, in adults. Therefore, the current environmental background exposure was unrelated to the tainted food exposures prior to the episode. This assumption was also supported by the comparisons of the participants' urinary DEHP metabolite measurements with those of other studies (S1 File, Section 6) and a follow-up study in Taiwan after the incident [25].

For follow-up epidemiology and health risk assessment, Table 3 compared the baseline characteristics of different exposure groups for the participants using a Wilcoxon rank-sum test for continuous variables and a chi-square test for categorical data. The results showed that children in the very high exposure group were relatively young with smaller mean age, BMI, and body weight. The percentage of maternal education level of college or above was also slightly lower than those of the other exposure groups. There was no significant difference for adolescents and adults.

**Table 3:** Baseline clinical characteristics of the participants of different exposure groups.

Participants Characteristics^a^		Exposure Group				
		Low	Medium	High	Very High	Overall
**Children**	N	85	83	34	25	227
	Sex (Male)	57.6%	57.8%	61.8%	64.0%	59.0%
	Age (Year)	6.8 (2.4)	5.4 (1.9)	5.8 (2.2)	4.7 (1.7)***	5.9 (2.4)
	BMI	17.2 (2.6)	15.7 (1.9)	15.6 (1.6)	15.5 (1.4)***	16.2 (2.2)
	Weight (Kg)	26.1 (9.3)	19.2 (5.1)	20.9 (8.0)	17.7 (3.4)***	21.9 (8.1)
	Birth weight (Gram)	3229 (468)	3050 (566)	3139 (566)	3070 (483)	3133 (521)
Maternal characteristics^b^						
	Age at pregnancy (Year)	30.8 (3.6)	31.0 (3.4)	30.4 (2.8)	29.7 (3.2)	30.7 (3.4)
	Smoking during pregnancy (Yes)	3.5%	2.4%	0	0	3.5
	Alcohol consumption during pregnancy (Yes)	3.6%	3.6%	0	0	3.0
	Type of delivery (Vaginal delivery or natural birth)	54.1%	56.6%	60.6%	80.0%	58.8%
	Education (College graduated or above)	84.7%	95.2%	87.9%	76.0%*	88.6%
**Adolescents**	N	9	2	1	0	12
	Sex (Male)	55.6%	100.0%	0	.	58.3%
	Age (Year)	14.3 (1.7)	12.1 (0.1)	15.6	.	14.1 (1.8)
	BMI	20.5 (2.3)	22.7 (5.0)	16.1	.	20.5 (3.6)
	Weight (Kg)	54.8 (11.5)	52.6 (4.8)	39.0	.	53.1 (13.1)
**Adults**	N	82	9	3	1	95
	Sex (Male)	42.7%	55.6%	33.3%	100%	44.2%
	Age (Year)	39.9 (9.8)	43.0 (9.3)	29.0 (6.7)	71.0	40.2 (10.3)
	BMI	25.8 (25.9)	24.1 (4.7)	20.7 (2.1)	17.1	25.4 (23.6)
	Weight (Kg)	64.5 (17.9)	66.8 (16.5)	56.9 (16.5)	46.1	64.3 (17.9)
	Smoke (Yes)	16.3%	25.0%	0	0	16.0%
	Alcohol Consumption (Yes)	6.3%	12.5%	0	0	6.4%
	Education (College graduated or above)	86.3%	87.5%	100%	0	84.0%
	Annual income (1.5 million NTD or above)	15.0%	12.5%	0	0	13.8%

^a^Characteristics presented by mean (standard deviation) for continuous variables or % for categorical variables

^b^ The pregnant women while expecting the children above

p-value:

*< 0.05

***< 0.001

### Strengths and Limitations

The DEHP dataset we analyzed in this study, using official information from the DOH and KBOH, is the most comprehensive to date since the incident [2,10,13,25]. Because of regular daily intake of the tainted nutritional supplements, many participants (213 out of 237 children and 74 out of 97 adults) provided detailed information on the product names, consumption amount, frequency, and duration. Using this complete information, we were able to estimate their AvDIs with much less uncertainty.

We classified the uncertainties of the participants' exposure to the tainted foods according to their responses to the study questionnaire and self-report. Different scenarios using Bayesian MCMC simulations were then adopted to estimate the daily intake depending on the degree of uncertainty on the exposure information. With the inherent advantages of Bayesian MCMC approach [26], the estimated AvDIs as a distribution from 10,000 MCMC simulations after convergence, rather than as point estimates, have the merit of reflecting all sources of uncertainties in the exposure assessment. The results showed that the estimated AvDIs had reasonable 95% CIs (Fig 4). As shown in S1 Fig, depending on the uncertainties on the concentration levels and the likelihood of being exposed, the estimated AvDIs had different distribution shapes resulting from the simulation outcomes. The developed methodology may also be applied to other exposure assessment that relies on questionnaires and self-report with various sources and degrees of uncertainties.

There are some limitations in this study. First, background contamination in foods during processing or packaging was undetected due to the screening level of 1 ppm. Because background exposures was taken into account in AvDI_ENV_ and the probability of *p*_1_ had been adjusted in our estimates, this should not be of much concern. Second, there was a possibility of recall bias owing to the time-lag of more than one year. Approximately 90% of the children and 76% of the adults provided detailed exposure histories on nutritional supplements, in contrast to occasional consumptions of the tainted drinks and fruit jams. Therefore, the contribution from recall bias of consumption behaviors of the tainted drinks and fruit jams should be minimal. We could not rule out the possibility of over-reporting, which could lead to overestimation [27,28]. We have reviewed the dataset for the participants' responses to the exposure questionnaire and their self-report: 11 children and 1 adult who had multiple supplement intakes simultaneously and/or with high consumption frequencies per week were considered likely over-reported. The exposure information for these participants were reconfirmed by telephone afterwards. Therefore, the consistency of the reported information should be reliable. However, 2 children who reported having consumption frequency of 28 times per week might still be over-report cases (estimated AvDI 224.6 ppm, 325.1 ppm, respectively). Also, 2 children (without self-report) with very high estimated AvDIs (310.0 ppm, 414.1ppm, respectively) due to having consumed the tainted juice beverages of high DEHP concentrations for a period of time might be over reported. A sensitivity analysis with and without these 4 children is suggested in assessing associations of the exposure estimates with health biomarkers.

Third, a large proportion of the participants were uncertain of their exposure to sport drinks, tea, juice, and fruit jams. Because of the low detection rates (0.01 to 0.04) in these food categories and the relatively low mean concentrations, except for in fruit jams, the effect should be relatively small. Fourth, single urinary DEHP metabolite measurement might not accurately reflect variations. As observed in a pharmacokinetic study from repeated blood and urine samples [29], potential bias resulting from single urine measurement should be minimal. Finally, it is possible that the bodyweights of the children might also be affected by DEHP exposure [30]. The adjusted bodyweight using eq (6), however, was unaffected by whether a child was obese owing to DEHP exposure.

In this study, DEHP exposure via contaminated food intake was reconstructed based on the developed Bayesian method by linking DEHP concentration data, participants' FFQ outcomes, self-reported exposure histories, and biomonitoring data. According to the results, a substantial proportion of the participants were exposed to DEHP at medium (25–50 *μg* / *kg*_*bw* / *day*) to high (≥50 *μg* / *kg*_*bw* / *day*) AvDIs prior to the episode, especially among the children. Further studies are necessary to assess the associations between the estimated exposure and the related health outcomes of these participants. Because of the commonly encountered uncertainty problems, the developed method can be used to retrospectively reconstruct exposure for epidemiological studies and quantitative health risk assessments.

## Supporting Information

S1 Fig Probability distribution plots of the estimated *AvDI*_*ALL*_ in children who were exposed to DEHP with a mean: a) low (<20 *μg* / *kg*_*bw* / *day*); b) medium (20–50 *μg* / *kg*_*bw* / *day*); c) high (50–100 *μg* / *kg*_*bw* / *day*); and d) very high (>100 *μg* / *kg*_*bw* / *day*) daily intake. Three children were randomly chosen from each of the exposure groups.
(TIF)

S1 File Supporting Information. (DOCX)

S1 Table Classifications of measured concentration intervals of the tainted foods for mixture distribution and the parameter estimates from MCMC simulations. (DOCX)

S2 Table Probability distributions used for consumption amount. (DOCX)

S3 Table Questionnaire outcomes of the participants according to the five food categories and whether they had provided self-reported exposure histories. (DOCX)

S4 Table Summary of estimated overall AvDIs of DEHP of the participants. (DOCX)

S5 Table Correlations between the reconstructed *AvDI*_*QN*_,*AvDI*_*SF*_, and the questionnaire scores. (DOCX)

S6 Table Summary of studies on urinary DEHP metabolite concentrations. (DOCX)

S7 Table Ratio of estimated background exposure to the overall daily intake of DEHP. (DOCX)

## References

[1] Wittassek M, Koch HM, Angerer J, Bruning T. Assessing exposure to phthalates—the human biomonitoring approach. Mol Nutr Food Res. 2011; 55:7–31. 10.1002/mnfr.201000121 20564479

[2] Yang J, Hauser R, Goldman RH. Taiwan food scandal: The illegal use of phthalates as a clouding agent and their contribution to maternal exposure. Food Chem Toxicol. 2013; 58: 362–368. 10.1016/j.fct.2013.05.010 23684997

[3] Chen ML, Chen JS, Tang CL, Mao IF. The internal exposure of Taiwanese to phthalate—An evidence of intensive use of plastic materials. Environ Int. 2008; 34: 79–85. 17765308 10.1016/j.envint.2007.07.004

[4] Hauser R, Duty S, Godfrey-Bailey L, Calafat AM. Medications as a source of human exposure to phthalates. Environ Health Perspect 2004; 112: 751–753. 15121520 10.1289/ehp.6804 PMC1241971

[5] Lyche JL, Gutleb AC, Bergman A, Eriksen GS, Murk AJ, Ropstad E, et al Reproductive and developmental toxicity of phthalates. J Toxicol Environ Health B. 2009; 12: 225–249. 10.1080/10937400903094091 20183522

[6] Jurewicz J, Hanke W. Exposure to phthalates: Reproductive outcome and children health. A review of epidemiological studies. Int J Occup Med Environ Health. 2011; 24(2): 115–141. 10.2478/s13382-011-0022-2 21594692

[7] Larsson M, Hägerhed-Engman L, Kolarik B, James P, Lundin F, Janson S, et al PVC—as floor material—and its association with incident asthma in a Swedish child cohort study. Indoor Air. 2010; 20: 494–501. 10.1111/j.1600-0668.2010.00671.x 21070375

[8] Halden RU. Plastics and health risks. Annu Rev Public Health. 2010; 31: 179–194. 10.1146/annurev.publhealth.012809.103714 20070188

[9] Wu MT, Wu CF, Chen BH, Chen EK, Chen YL, Shiea J, et al Intake of phthalate-tainted foods alters thyroid functions in Taiwanese children. PLoS ONE. 2013; 8(1): e55005 10.1371/journal.pone.0055005 23383031 PMC3559382

[10] Lin LC, Wang SL, Chang YC, Huang PC, Cheng JT, Su PH, et al Associations between maternal phthalate exposure and cord sex hormones in human infants. Chemosphere. 2011; 83(8): 1192–1199. 10.1016/j.chemosphere.2010.12.079 21272909

[11] Wittassek M, Heger W, Koch HM, Becker K, Angerer J, Kolossa-Gehring M. Daily intake of di(e-ethylhexyl)phthalate (DEHP) by German children—A comparison of two estimation models based on urinary DEHP metabolite levels. Int J Hyg Environ Health. 2007; 35–42. 17185035 10.1016/j.ijheh.2006.11.009

[12] Fierens T, Servaes K, Van Holderbeke M, Geerts L, De Henauw S, Sioen I, et al Analysis of phthalates in food products and packaging materials sold on the Belgian market. Food Chem Toxicol. 2012; 50: 2575–2583. 10.1016/j.fct.2012.04.029 22554646

[13] Wu MT, Wu CF, Wu JR, Chen BH, Chen EK, Chao MC, et al The public health threat of phthalate-tainted foodstuffs in Taiwan: The policies the government implemented and the lessons we learned. Environ Int. 2012; 44: 75–79. 10.1016/j.envint.2012.01.014 22361240

[14] Yen TH, Lin-Tan DT, Lin JL. Food safety involving ingestion of foods and beverages prepared with phthalate-plasticizer-containing clouding agents. J Formos Med Assoc. 2011; 110: 671–684. 10.1016/j.jfma.2011.09.002 22118310

[15] Lorber M, Angerer J, Koch HM. A simple pharmacokinetic model to characterize exposure of Americans to Di-2-ethylhexyl phthalate. J Exp Sci Environ Epi. 2010; 20: 38–53. 10.1038/jes.2008.74 19127283

[16] Hines CJ, Hope NB, Deddens JA, Silva MJ, Calafat AM. Estimated daily intake of phthalates in occupationally exposed groups. J Exp Sci Environ Epi. 2011; 21: 133–141. 10.1038/jes.2009.62 20010977

[17] Schecter A, Lorber M, Guo Y, Wu Q, Yun SH, Kannan K, et al Phthalate concentrations and dietary exposure from food purchased in New York State. Environ Health Perspect. 2013; 121(4): 473–479. 10.1289/ehp.1206367 23461894 PMC3620091

[18] Sakhi AK, Lillegaard ITL, Voorspoels S, Carlsen MH, Løken EB, Brantsæter AL, et al Concentrations of phthalates and bisphenol A in Norwegian foods and beverages and estimated dietary exposure in adults. Environ Int. 2014; 73: 259–269. 10.1016/j.envint.2014.08.005 25173060

[19] Fierens T, Servaes K, Van Holderbeke M, Geerts L, De Henauw S, Sioen I, et al Analysis of phthalates in food products and packaging materials sold on the Belgian market. Food Chem Toxicol. 2012; 50: 2575–2583. 10.1016/j.fct.2012.04.029 22554646

[20] Huang PC, Tsai CH, Liang WY, Li SS, Pan WH, Chiang HC. Age and gender differences in urinary levels of eleven phthalate metabolites in general Taiwanese population after a DEHP episode. PLoS ONE. 2015;10(7): e0133782 10.1371/journal.pone.0133782 26207744 PMC4514596

[21] Huang HB, Chen HY, Su PH, Huang PC, Sun CW, Wang CJ, et al Fetal and childhood exposure to phthalate diesters and cognitive function in children up to 12 years of age: Taiwanese Maternal and Infant Cohort Study. PLoS ONE. 2015; 10(6):e0131910 10.1371/journal.pone.0131910 26121592 PMC4488303

[22] Remer T, Neubert A, Maser-Gluth C. Anthropometry-based reference values for 24-h urinary creatinine excretion during growth and their use in endocrine and nutritional research. Am J Clin Nutr. 2002; 75: 561–569. 11864864 10.1093/ajcn/75.3.561

[23] Anderson WAC, Castle L, Hird S, Jefferey J, Scotter MJ. A twenty-volunteer study using deuterium labelling to determine the kinetics and fractional excretion of primary and secondary urinary metabolites of di-2-ethylhexylphalate and di-iso-nonylphthalate. Food Chem Toxicol. 2011; 49: 2022–2029. 10.1016/j.fct.2011.05.013 21609750

[24] Chen W, Chang MH. New growth charts for Taiwanese children and adolescents based on world health organization standards and health-related physical fitness. Pediatr Neonatol. 2010; 51(12): 69–79. 20417456 10.1016/S1875-9572(10)60014-9

[25] Wu CF, Chen BH, Shiea J, Chen EK, Liu CK, Chao MC, et al Temporal changes of urinary oxidative metabolites of di(2-ethlhexyl)phthalate after the 2011 phthalate incident in Taiwanese children- Findings of a 6-month follow-up. Environ Sci Technol. 2013; 47: 13754–13762. 10.1021/es403141u 24191740

[26] Gilks WR, Richardson SR, Spiegelhalter DJ. Markov Chain Monte Carlo in Practice. Chapman & Hall/CRC: Boca Rato, Florida; 1996.

[27] Piro FN, Madsen C, Næss Ø, Nafstad P, Claussen B. A comparison of self reported air pollution problems and GIS-modled levels of air pollution in people with and without chronic diseases. Environ Health. 2008; 7:9 10.1186/1476-069X-7-9 18307757 PMC2289819

[28] Brauer C, Mikkelsen S. The influence of individual and contextual psychosocial work factors on the perception of the indoor environment at work: a multilevel analysis. Int Arc Occup Environ Health. 2010; 83(6): 639–651. 10.1007/s00420-010-0511-9 20143083

[29] Lorber M, Koch HM. Development and application of simple pharmacokinetic models to study human exposure to di-n-butyl phthalate (DnBP) and diisobutyl phthalate (DiBP). Environ Int. 2013; 59: 469–477. 10.1016/j.envint.2013.07.010 23955327

[30] Kim SH, Park MJ. Phthalate exposure and childhood obesity. Ann Pediatr Endocrinol Metab. 2014; 19: 69–75. 10.6065/apem.2014.19.2.69 25077088 PMC4114051

