# Supplementary figures and images for: Exposure Estimation for Risk Assessment of the Phthalate Incident in Taiwan
Source: PLoS One. 2016 Mar 9;11(3):e0151070. doi: 10.1371/journal.pone.0151070 (PMC4784747; doi:10.1371/journal.pone.0151070)

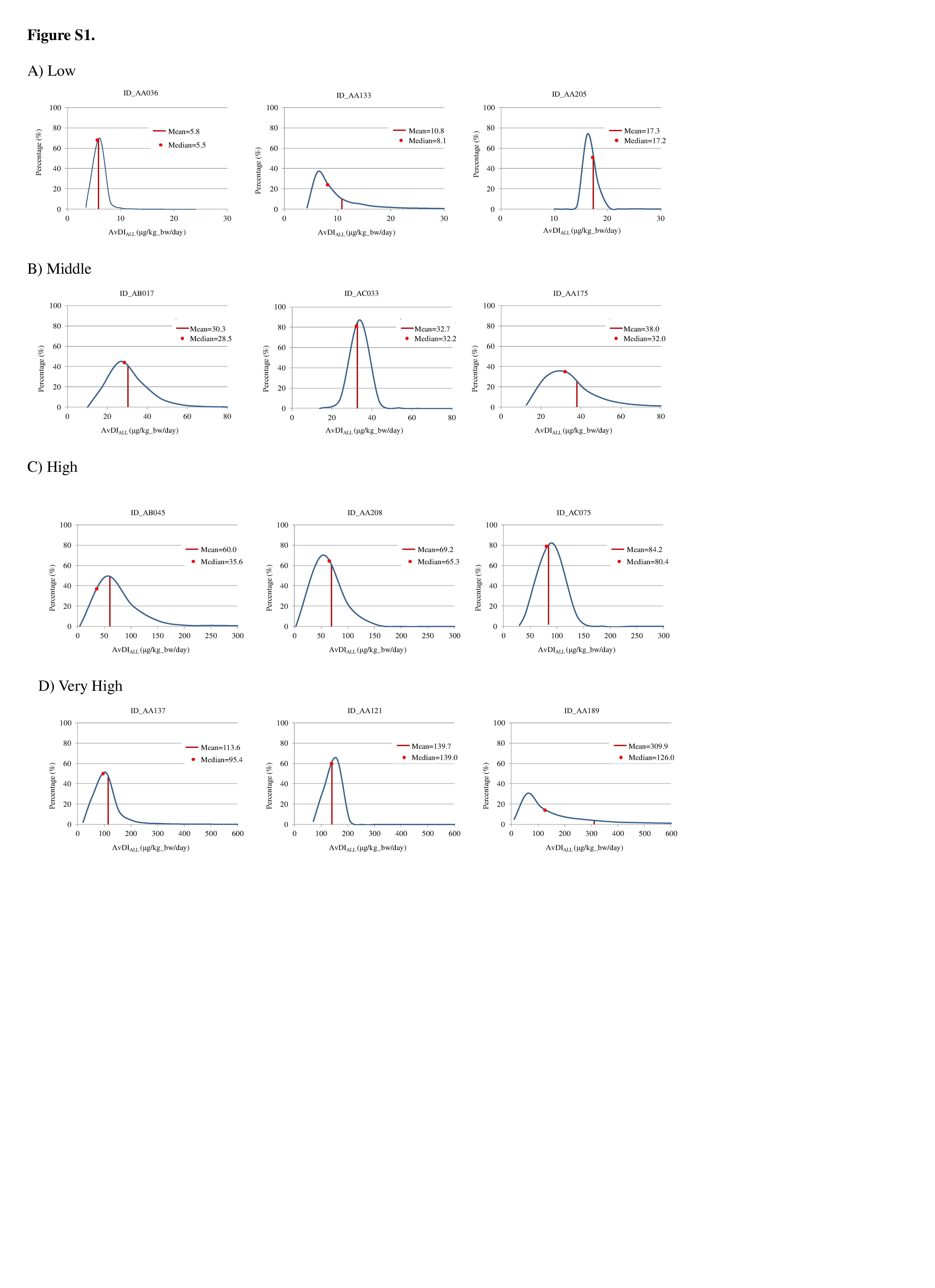

Supplement: S1 Fig — Three children were randomly chosen from each of the exposure groups. (TIF) [file pone.0151070.s001.tif]
