# Supplementary material for: Exposure Estimation for Risk Assessment of the Phthalate Incident in Taiwan
Source: PLoS One. 2016 Mar 9;11(3):e0151070. doi: 10.1371/journal.pone.0151070 (PMC4784747; doi:10.1371/journal.pone.0151070)
Supplement: S1 File — (DOCX) [file pone.0151070.s002.docx]

**S1 File**

**Supporting Information**

Chu-Chih Chen^1*^, Shu-Li Wang^2^, Ming-Tsang Wu^3,4,5^, Yin-Han Wang^1^, Po-Chin Huang^2^, Bai-Hsiun Chen^6,7^, Chien-Wen Sun^2^, Chi-Kung Ho^8,9,10^, Yang-Chih Shih^11^, Ming-Neng Shiu^11^, Wen-Harn Pan^12,13^, Mei-Lien Chen^14^, Ching-Chang Lee^15,16^, Chao A. Hsiung^1*^, the RAPIT Group

^1^Division of Biostatistics and Bioinformatics, Institute of Population Health Sciences,

National Health Research Institutes, Miaoli, Taiwan

^2^National Institute of Environmental Health Sciences, National Health Research Institutes, Miaoli, Taiwan

^3^Department of Public Health, College of Health Sciences, Kaohsiung Medical University, Kaohsiung, Taiwan

^4^Department of Family Medicine, Kaohsiung Medical University Hospital, Kaohsiung, Taiwan

^5^Center of Environmental and Occupational Medicine, Kaohsiung Municipal Hsiao-Kang Hospital, Kaohsiung, Taiwan

^6^Department of Laboratory Medicine and Pediatrics, Kaohsiung Medical University Hospital, Kaohsiung Medical University, Kaohsiung, Taiwan

^7^Graduate Institute of Clinical Medicine, College of Medicine, Kaohsiung Medical University, Kaohsiung, Taiwan

^8^Department of Public Health, Kaohsiung Medical University, Kaohsiung, Taiwan

^9^Department of Occupational and Environmental Medicine, Kaohsiung Medical University Hospital, Kaohsiung, Taiwan

^10^Department of Health, Kaohsiung City Government, Kaohsiung, Taiwan

^11^Ministry of Health and Welfare, Taipei, Taiwan

^12^Division of Preventive Medicine and Health Services Research, Institute of Population Health Sciences, National Health Research Institutes, Miaoli, Taiwan

^13^Institute of Biomedical Sciences, Academia Sinica, Taipei, Taiwan

^14^Institute of Environmental and Occupational Health Sciences, College of Medicine, National Yang Ming University, Taipei, Taiwan

^15^Department of Environmental and Occupational Health, College of Medicine, National Cheng Kung University, Tainan, Taiwan

^16^Research Center of Environmental Trace Toxic Substance, National Cheng Kung University, Tainan, Taiwan

**Table of Contents**

1. List of health examinations and biomarker measurements for the study participants.............................................................................................................3
2. Simulation parameters for concentration mixture distribution of the DEHP- tainted foods and exposure questionnaire outcomes..............................................4
3. Calculation of consumption amount for average daily intake of DEHP................5
4. Uncertainties in DEHP concentrations and dietary intake exposures....................5
5. Correlations between the exposure scores and the reconstructed AvDIs...............7
6. Comparisons of the participants' background urinary DEHP metabolite concentrations with those of other studies.............................................................8

**1. List of physical examinations and biomarker measurements for the study participants**

The complete list of physical examinations and biomarker measurements held during the participant's clinic visit by the RAPIT are as follows:

**Physical examinations**. body weight, height, waist circumference, body fat composition, blood pressure, pubertal (Tanner) stage (children and adolescents), sperm quality (male adult), abdominal ultrasound, bone age, anogenital distance (male children and adolescents).

**Neurodevelopment assessment tests in children**. Bayley Scale of Infant Development (BSID-III), Wechsler Preschool and Primary Scale of Intelligence- Revised (WPPSI-R) for 3-6 years old children, Wechsler Intelligence Scale for Children- IV (WISC-IV) for 6-12 years old children, Wechsler Adult Intelligence Scale- 3rd Edition (WAIS-III).

**Biomarker measurements**. The endocrine profile markers: thyroid stimulating hormone (TSH), serum triiodothyronine (T3), thyroxine (T4), free T4, TBG, TTR, estradiol (E2), follicle stimulating hormone (FSH), luteinizing hormone (LH), testosterone (TT), free TT, albumin, sex hormone binding globulin, dehydroepiandrosterone sulfate (DHEA-S), prolactin (female adult), inhibin B, progesterone (PG), anti-mullerian hormone (AMH), dihydrotesterone, estrone (E1);liver and kidney functions in serum: glutamate oxaloacetate transaminase (GOT), glutamate pyrurate transaminase (GPT), gamma-glutamyl transpeptidase (GGT), uric acid (UA), blood urea nitrogen (BUN), blood creatinine, urine creatinine; immune function markers: immunoglobulin E (IgE), IgG; cardiovascular markers: glycated hemoglobin (HbA1c), sugar AC, lipid profile including triglyceride (TG), PG, high-density lipoprotein cholesterol (HDL-C), low-density lipoprotein cholesterol (LDL-C); cancer markers: carcinoembryonic antigen (CEA), alphafetaprotein (AFP), carbohydrate antigen (CA) 19-9, Ca153, Ca125, prostatic specific antigen (PSA); blood lead level; blood and urine examinations.

**2. Simulation parameters for concentration mixture distribution of the DEHP-contaminated foods and exposure questionnaire outcomes**

Table S1 summarizes the classification of the detected concentration intervals, for the five contaminated-food categories, that were used for the calculations of their mixture distribution. The weight parameter, $\pi$, and resultant parameter estimates, $\mu$ and $\sigma$, of the lognormal distribution from MCMC simulations are also listed in Table S1.

Table S2 summarizes the reported exposure scenarios for the participants according to their questionnaire outcomes and their self-reported exposure history to DHEP-contaminated foods. Of the five food categories, exposure to health or nutritional supplements in children yielded the most complete data. Approximately 80% (191 out of 237) of the children were confirmed to have been exposed to contaminated supplements for a substantial period because of daily consumption. The exposure history for a total of 213 children was provided by their caregivers. However, 158 of these had incomplete exposure information, with either unknown product name(s), intake amount, or consumption frequency. In contrast, only 12, 0, 4, and 8 children responded that they had consumed contaminated sport drinks, tea drinks, juice beverages, and fruit jams, respectively, and 36, 60, 55, and 56, respectively, were uncertain of their exposure to these contaminated products. Most of the children had consumption frequencies for these food categories of less than twice per week and, thus, the corresponding AvDIs of DEHP were excluded. Similarly, 67 (out of 97) adults responded that they had taken contaminated health or nutrition supplements, in contrast to only 15, 2, 4, and 6 who reported exposure to contaminated sport drinks, tea drinks, juice beverages, and fruit jams, respectively.

**3. Calculation of consumption amount for average daily intake of DEHP**

The consumption volume of sport drinks, tea drinks, and juice beverages for children and for adolescents and adults were assumed to be 350 ml, 250 ml, and 250 ml; and 350 ml, 500 ml, and 500 ml per time, respectively. Because of uncertain variations in fruit/jams/nectar/jelly, the consumption amount of fruit jams/nectar per time was simulated from a normal distribution with a mean of 2.5 grams. The consumption amount of nutrition supplements was determined from participants' self-report of consumption dosage per time of specific product, and was simulated from a normal distribution for the participants who did not provide a self-report of consumption dosage. The means of simulated normal distribution were estimated from those participants who provided the related information. See Table S3 for details of the parameter settings.

**4. Uncertainties in DEHP concentrations and dietary intake exposures**

**Uncertainty regarding the DEHP concentrations in the tainted food products**

A probabilistic approach was adopted to describe the variation and/or uncertainties of the DEHP concentrations in the tainted foods. The DEHP concentration in a specific tainted food product with a single measurement was described by a lognormal distribution,$\log N\left(\mu,\sigma^{2}\right)$, with the measurement defined as the mean $\mu$ and the standard deviation $\sigma$ defined by a coefficient variation of 0.1 times the mean. For a specific food product with multiple measurements, the concentration was described by a lognormal distribution,$\log N\left(\mu,\sigma^{2}\right)$, with the mean and variance estimated from the multiple measurements. For the tainted food products without a measurement, a mixture distribution [S1] was used to describe the unknown DEHP concentration *Y*, which is proportional in probability to the distributions of the food products within the same category, i.e.,

$Y\sim\sum_{k=1}^{K} \pi_{k}\log N\left(\mu_{k},\sigma_{k}^{2}\right)$, (S1)

where the relative weight $\pi_{k}$ is the proportion of the tainted foods within the *k*-th concentration interval, $k=1,\cdots,K$. The food products with single measurement within the same interval were grouped together in counting $\pi_{k}$. The tainted foods with multiple measurements constituted separate lognormal distributions, with the weights being one over the number of the food products in the category. A classification of the concentration intervals for each food category is given in Table S1.

For food products in which DEHP was undetected (<1 ppm), the actual DEHP concentration was either 0 ppm or between 0 ppm and 1 ppm. Let $p_{ND}$ be the probability of being undetected, then we have

$p_{ND}=p_{0}+\left(1-p_{0}\right)p_{1}$, (S2) where $p_{0}$ and $p_{1}$ are the probabilities of being 0 ppm and between 0 ppm and 1 ppm, respectively. Because the TFDA specifically targeted the food products that could be contaminated, there was a sampling bias towards higher detection rates that need to be corrected. The non-detection probability, $p_{ND}$, for each of the food categories was estimated using a logistic model:

$\log p_{ND}/\left(1-p_{ND}\right)=\alpha+\beta*I\left\{TFDA\right\}$, (S3)

where $I\left\{TFDA\right\}$ is the indicator function such that $I\left\{TFDA\right\}=1$ if the sample was from TFDA and 0 otherwise. The odds ratio for the non-detection rate of TFDA over that of the KBOH was$\exp\left(\beta\right)$.

A Bayesian statistical procedure using MCMC simulation was employed to estimate the distribution parameters of the DEHP concentration in the contaminated foods for each category. The probability $p_{1}$ was estimated using the proportion of the 10,000 simulated concentrations that were below 1 ppm. The probability $p_{0}$ was then obtained using eqs (S2) and (S3).

**Uncertainties regarding to participants' exposure to DEHP through dietary intakes**

Based on the participants' responses to the study questionnaire and self-report, participant exposure scenarios were classified into different scenarios for exposure reconstruction. If a participant provided the name(s) of a specific food product(s) with measurement(s), then the exposure concentration Y was generated from the lognormal distribution for that food product. If the name of the tainted food product was unknown or there was no measurement, then *Y* was generated from the constructed mixture distribution (eq S1). If a participant was not sure of having been exposed, then *E(C)* was used for concentration *Y* [S2]:

$E\left(C\right)=p_{0}*0+\left(1-p_{0}\right)*E\left(Y\right)$, (S4)

where *E(Y)* is the mean concentration of the mixture distribution. Because juice beverages are made from concentrated fruit jams and nectar, the DEHP concentrations in this food category were divided by a factor *d* of normal distribution with a mean of 7 and variance of 1 [S3].

**5.** **Correlations between the exposure scores and the reconstructed AvDIs**

To assess the consistency of the questionnaire outcomes and the exposure reconstruction, Spearman correlation coefficients were calculated for the Q1-Q5 questionnaire scores and the reconstructed AvDIs for children, adolescents, and adults separately. Table S5 summarizes the results. The correlations between $AvDI_{QN}$ and the exposure scores for each of the contaminated food categories were highly significant, except for the juice beverage category among children, with most of the estimated AvDIs clustered at a concentration of approximately 0 ppm. Additionally, $AvDI_{SF}$ and $AvDI_{QN}$ were significantly correlated in children for all the food categories combined and for the supplements alone. Similarly, in adults, the correlation between $AvDI_{SF}$ and $AvDI_{QN}$ was greater than the correlation between $AvDI_{SF}$ and the exposure scores. Except for the Q3 score for juice beverage in children, all of the reconstructed AvDIs (*AvDI_QN1_*- *AvDI_QN5_*) correlated significantly with the corresponding questionnaire score for the food category. Similarly, the AvDI_QN_ correlated significantly with the Q1-Q5 summary scores in adults. The correlations were even higher when only the health or nutrition supplements were considered. For the pairwise correlations, AvDI_SF_ was significantly correlated with AvDI_QN_ in children, however, it did not correlate significantly with the Q1-Q5 summary scores either in children or in adults. The results showed that the estimated AvDIs, either from the exposure questionnaire or the self-reported exposure history, had an overall higher association with each other than would be observed using the score method.

**6. Comparisons of the participants' background urinary DEHP metabolite**

**concentrations with those of other studies**

Table S6 summarizes the urinary DEHP metabolite concentration levels observed in the current study and those from other studies held previously in Taiwan [S4,S5] and worldwide [S6-S10]. The mean concentrations of the metabolites MEHP, MEOHP, MEHHP (13.1 $\mu g/g$ $Cr$, 40.8 $\mu g/g$ $Cr$, and 58.4 $\mu g/g$ $Cr$, respectively) in children of this study were close to those observed in a German study [6], and were several times higher than the levels observed in adolescents and adults in this study. Compared to values from studies conducted in Taiwan prior to the incident [S4,S5], the mean urinary metabolite concentration levels in this study were approximately the same or two to three times lower. However, though comparable with other Asian countries [S6], the metabolite concentration levels of the participants were still several times higher than those found in the U.S. and European countries [S7-S10]. Because the study participants were from a potentially highly exposed population, the results appeared to be reasonable in comparison with other studies targeting the general population [S4,S5]. To compare the participants' background exposure levels with the estimated exposure from intake of contaminated foods, we also calculated the ratio of AvDI_ENV_ over AvDI_ALL_ (Table S7). The results showed that the median ratios ranged from 0.54 to 0.05 in children and 0.32 to 0.02 in adults from the low exposure group (<20 $\mu g/kg\_bw/day$) to the very high exposure group (>100 $\mu g/kg\_bw/day$). Thus, for a highly exposed participant, the exposure to DEHP from contaminated foods would be at least twenty-fold higher than background DEHP exposure.

**References**

1. Lindsay BG. Mixture Models: Theory, Geometry and Applications. Institute of Mathematical Statistics; 1995.
2. Kennedy MC. Bayesian modeling of long-term dietary intakes from multiple sources. Food Chem Toxicol. 2010; 48: 250–263.
3. Cool Concentrated Juice Series. Available: http://www.pcstore.com.tw/hecto-coffee/M04020935.htm. (In Chinese)
4. Lin S, Ku HY, Su PH, Chen JW, Huang PC, Angerer J, et al. Phthalate exposure in pregnant women and their children in central Taiwan. Chemosphere. 2011;82: 947–955.
5. Hsu NY, Lee CC, Wang JY, Li YC, Chang HW, Chen CY, et al. Predicted risk of childhood alergy, asthma, and reported symptoms using measured phthalate exposure in dust and urine. Indoor Air. 2012;22:186–199.
6. Guo Y, Alomirah H, Cho HS, Minh TB, Mohd MA, Nakata H, et al. Occurrence of phthalate metabolites in human urine from several Asian countries. Environ Sci Technol*.* 2011;45:3138–3144.
7. Becker K, Seiwert M, Angerer J, Heger W, Koch HM, Nagorka R, et al. DEHP metabolites in urine of children and DEHP in house dust. Int J Hyg Environ Health. 2004;207:409–417.
8. Boas M, Frederiksen H, Feldt-Rasmussen U, Skakkerbæk NE, Hegedüs L, Hilsted L, et al. Childhood exposure to phthalates: Associations with thyroid function, insulin-like growth factor I, and Gowth. Environ Health Perspect*.* 2010; 118:1458–1464.
9. Kasper-Sonnenberg M, Koch HM, Wittsiepe J, Wilhelm M. Levels of phthalate metabolites in urine among mother—child-pairs—Results from the Duisburg birth cohort study, Germany. Int J Hyg Environ Health. 2012;215:373–382.
10. Fourth National Report on Human Exposure to Environmental Chemicals, February 2015, US CDC. Available:

<http://www.cdc.gov/biomonitoring/pdf/FourthReport_UpdatedTables_Feb2015.pdf>.
