# Supplementary material for: Exposure Estimation for Risk Assessment of the Phthalate Incident in Taiwan
Source: PLoS One. 2016 Mar 9;11(3):e0151070. doi: 10.1371/journal.pone.0151070 (PMC4784747; doi:10.1371/journal.pone.0151070)
Supplement: S1 Table — (DOCX) [file pone.0151070.s003.docx]

**Table S1.**

| **Food category** | **DEHP concentration range (ppm)** | **Concentration record** | | π | μ^b^ | σ^b^ |
| --- | --- | --- | --- | --- | --- | --- |
|  |  | **Single** | **Multiple^a^** |  |  |  |
| Sport drinks | 1-40 | 2 |  | 0.5 | 2.16 | 0.96 |
|  |  |  | (5) | 0.25 | 2.6 | 0.04 |
|  |  |  | (4) | 0.25 | 2.4 | 0.1 |
| Tea drinks | 1-100 | 3 |  | 1 | 2.38 | 1.09 |
| Juice beverages | 1-100 | 9 |  | 0.9 | 2.59 | 1.46 |
|  |  |  | (3) | 0.1 | 2.15 | 0.58 |
| Fruit jam, nectar, or jelly | 1-100 | 3 |  | 0.3 | 2.31 | 1.23 |
|  | 100-500 | 7 |  | 0.7 | 3.80 | 1.70 |
| Health or nutrition supplements | 1-10 | 65 |  | 0.516 | 1.19 | 0.58 |
|  |  |  | (2) | 0.008 | 1.92 | 0.21 |
|  |  |  | (2) | 0.008 | 2.07 | 0.15 |
|  |  |  | (2) | 0.008 | 2.01 | 0.03 |
|  |  |  | (2)^c^ | 0.008 | 0.13 | 0.49 |
|  |  |  | (2)^c^ | 0.008 | 1.52 | 0.86 |
|  |  |  | (2) | 0.008 | 0.94 | 0.19 |
|  | 10-50 | 20 |  | 0.159 | 2.38 | 0.95 |
|  |  |  | (2) | 0.008 | 3.52 | 0.05 |
|  | 50-100 | 8 |  | 0.063 | 2.95 | 1.27 |
|  |  |  | (2) | 0.008 | 4.34 | 0.18 |
|  |  |  | (2) | 0.008 | 4.43 | 0.10 |
|  |  |  | (2) | 0.008 | 3.84 | 0.68 |
|  |  |  | (2) | 0.008 | 3.66 | 1.27 |
|  | 100-300 | 10 |  | 0.079 | 3.45 | 1.51 |
|  |  |  | (3) | 0.008 | 5.31 | 0.53 |
|  |  |  | (2) | 0.008 | 4.86 | 0.60 |
|  |  |  | (2) | 0.008 | 4.27 | 1.13 |
|  | 300-1000 | 4 |  | 0.032 | 4.21 | 1.81 |
|  |  |  | (2) | 0.008 | 6.15 | 0.40 |
|  |  |  | (3) | 0.008 | 5.74 | 0.76 |
|  |  |  | (2) | 0.008 | 5.89 | 0.45 |
|  | > 1000 | 1 |  | 0.008 | 4.73 | 1.83 |
|  |  |  | (3) | 0.008 | 7.35 | 0.56 |
| ^a^ The number in the parenthesis is the records of DEHP concentration measurements of the specific food product.  ^b^μ and σ are the location and scale parameter estimates of the corresponding lognormal distribution from MCMC simulations.  ^c^One of the DEHP concentrations was < 1 ppm and was replaced by an estimate of 0.5 ppm. | | | | | | |
