# Supplementary material for: Exposure Estimation for Risk Assessment of the Phthalate Incident in Taiwan
Source: PLoS One. 2016 Mar 9;11(3):e0151070. doi: 10.1371/journal.pone.0151070 (PMC4784747; doi:10.1371/journal.pone.0151070)
Supplement: S2 Table — (DOCX) [file pone.0151070.s004.docx]

**Table S2.**

|  |  | **Children** | | |  | **Adolescents** | | |  | **Adults** | | |
| --- | --- | --- | --- | --- | --- | --- | --- | --- | --- | --- | --- | --- |
|  |  | **Not sure** | **No** | **Yes** |  | **Not sure** | **No** | **Yes** |  | **Not sure** | **No** | **Yes** |
| Sport drinks | Frequency >= 2 per week? |  | 153 | 84 |  |  | 10 | 3 |  |  | 50 | 47 |
|  | Had consumed the contaminated sport drinks? | 36 | 36 | 12 |  | 3 | 0 | 0 |  | 22 | 10 | 15 |
|  | Provided self-report? |  | 235 | 2 |  |  | 13 | 0 |  |  | 86 | 11 |
|  | Detailed exposure history? |  | 0 | 2 |  |  | 0 | 0 |  |  | 5 | 6 |
| Tea drinks | Frequency >= 2 per week? |  | 134 | 103 |  |  | 10 | 3 |  |  | 32 | 65 |
|  | Had consumed the contaminated tea drinks? | 60 | 43 | 0 |  | 3 | 0 | 0 |  | 52 | 11 | 2 |
|  | Provided self-report? |  | 237 | 0 |  |  | 13 | 0 |  |  | 97 | 0 |
|  | Detailed exposure history? |  | 0 | 0 |  |  | 0 | 0 |  |  | 0 | 0 |
| Juice beverages | Frequency >= 2 per week? |  | 142 | 95 |  |  | 10 | 3 |  |  | 53 | 44 |
|  | Had consumed the contaminated juice beverage? | 55 | 36 | 4 |  | 2 | 0 | 1 |  | 32 | 8 | 4 |
|  | Provided self-report? |  | 236 | 1 |  |  | 13 | 0 |  |  | 93 | 4 |
|  | Detailed exposure history? |  | 0 | 1 |  |  | 0 | 0 |  |  | 4 | 0 |
| Fruit jam, nectar or jelly | Frequency >= 2 per week? |  | 156 | 81 |  |  | 9 | 4 |  |  | 63 | 34 |
|  | Had consumed the DEHP-contaminated fruit jam, nectar or jelly? | 56 | 17 | 8 |  | 3 | 0 | 1 |  | 17 | 11 | 6 |
|  | Provided self-report? |  | 237 | 0 |  |  | 13 | 0 |  |  | 97 | 0 |
|  | Detailed exposure history? |  | 0 | 0 |  |  | 0 | 0 |  |  | 0 | 0 |
| Health or nutrition supplements | Frequency >= 2 per week? |  | 28 | 209 |  |  | 1 | 12 |  |  | 13 | 84 |
|  | Had taken the contaminated supplements? | 18 | 0 | 191 |  | 1 | 0 | 11 |  | 14 | 3 | 67 |
|  | Provided self-report? |  | 24 | 213 |  |  | 1 | 12 |  |  | 23 | 74 |
|  | Detailed exposure history? |  | 158 | 55 |  |  | 11 | 1 |  |  | 61 | 13 |
