# Supplementary material for: Exposure Estimation for Risk Assessment of the Phthalate Incident in Taiwan
Source: PLoS One. 2016 Mar 9;11(3):e0151070. doi: 10.1371/journal.pone.0151070 (PMC4784747; doi:10.1371/journal.pone.0151070)
Supplement: S3 Table — (DOCX) [file pone.0151070.s005.docx]

**Table S3.**

| **Consumption amount of fruit jams/nectar/jelly and nutrition supplements each time** | **Participants** | **Distribution** | **Mean ($\mu$)** | **Std ($\sigma$)** |
| --- | --- | --- | --- | --- |
| Fruit jams/nectar/jelly (g) | All | Normal | 2.5 g | 0.6 g |
| Nutrition supplements in the form of powder (g) | Children | Normal | 1.9 g | 0.5 g |
| Nutrition supplements in the form of powder (g) | Adolescents/adults | Normal | 2.7 g | 0.7 g |
| Nutrition supplements in the form of tablet/capsule (g) | Children | Normal | 1.0 g | 0.2 g |
| Nutrition supplements in the form of tablet/capsule (g) | Adolescents/adults | Normal | 1.6 g | 0.4 g |
| Nutrition supplements (form unknown) (g) | Children | Normal | 1.6 g | 0.23 g |
| Nutrition supplements (form unknown) (g) | Adolescents/adults | Normal | 2.0 g | 0.33 g |
| Dilution factor of juice beverages made from fruit jams/nectar/jelly | Children | Normal | 7 | 1 |
|  | | | | |
