# Supplementary material for: Exposure Estimation for Risk Assessment of the Phthalate Incident in Taiwan
Source: PLoS One. 2016 Mar 9;11(3):e0151070. doi: 10.1371/journal.pone.0151070 (PMC4784747; doi:10.1371/journal.pone.0151070)
Supplement: S4 Table — (DOCX) [file pone.0151070.s006.docx]

**Table S4.**

|  |  |  |  | **Percentile** | | | | | | |
| --- | --- | --- | --- | --- | --- | --- | --- | --- | --- | --- |
| **AvDI_ALL_**  (μg/kg_bw/day) |  | **Mean** | **SD** | **Min** | **5%** | **25%** | **50%** | **75%** | **95%** | **Max** |
| Without window period | Children | 46.4 | 56.1 | 3.4 | 6.1 | 14.8 | 29.2 | 51.5 | 152.0 | 414.1 |
|  | Adolescents | 17.6 | 20.9 | 1.4 | 1.4 | 7.7 | 10.0 | 21.0 | 76.9 | 76.9 |
|  | Adults | 12.3 | 17.8 | 1.1 | 1.5 | 3.8 | 6.8 | 12.8 | 46.0 | 126.4 |
| With window period | Children | 24.3 | 30.6 | 0.8 | 4.5 | 9.5 | 16.0 | 26.3 | 66.8 | 283.2 |
|  | Adolescents | 7.4 | 5.8 | 1.2 | 1.2 | 2.5 | 6.8 | 9.7 | 21.4 | 21.4 |
|  | Adults | 6.5 | 7.2 | 1.0 | 1.4 | 2.7 | 4.4 | 7.2 | 24.5 | 39.4 |
