# Supplementary material for: Exposure Estimation for Risk Assessment of the Phthalate Incident in Taiwan
Source: PLoS One. 2016 Mar 9;11(3):e0151070. doi: 10.1371/journal.pone.0151070 (PMC4784747; doi:10.1371/journal.pone.0151070)
Supplement: S5 Table — (DOCX) [file pone.0151070.s007.docx]

**Table S5.**

| **Exposure score** | | | | | | | |
| --- | --- | --- | --- | --- | --- | --- | --- |
|  |  | **Q1** | **Q2** | **Q3** | **Q4** | **Q5** | **Q1-Q5** |
| Children | n | 48 | 60 | 59 | 64 | 209 | 224 |
|  | AvDI_QN_ | 0.75*** | 0.51*** | 0.07 | 0.51*** | 0.47*** | 0.37*** |
|  | AvDI_SF_ |  |  |  |  |  | -0.002 |
|  | AvDI_env_ |  |  |  |  |  |  |
| Adolescents | n |  |  | 3 | 4 | 12 | 12 |
|  | AvDI_QN_ |  |  | 0.5 | 0.77 | 0.45 | 0.28 |
|  | AvDI_SF_ |  |  |  |  |  | -0.11 |
|  | AvDI_env_ |  |  |  |  |  |  |
| Adults | n | 37 | 54 | 36 | 23 | 81 | 95 |
|  | AvDI_QN_ | 0.86*** | 0.77*** | 0.79*** | 0.85*** | 0.82*** | 0.39*** |
|  | AvDI_SF_ |  |  |  |  |  | 0.11 |
|  | AvDI_env_ |  |  |  |  |  |  |
| ^a^AvDI_QN_, AvDI_SF_, and AvDI_env_ are the average daily dose of DEHP estimated from exposure assessment questionnaire, self-reported exposure history, and background environmental exposure to DEHP estimated from metabolite concentrations in urine.  ^b^The Spearman correlation coefficients between AvDI_SF_ and AvDI_QN_ in children and adults were 0.23 (p-value 0.001, n=201) and 0.16 (p-value 0.15, n=81), respectively.  ^c^* ,P<0.05; **, P<0.01;***, P<0.001. | | | | | | | |
