# Supplementary material for: Exposure Estimation for Risk Assessment of the Phthalate Incident in Taiwan
Source: PLoS One. 2016 Mar 9;11(3):e0151070. doi: 10.1371/journal.pone.0151070 (PMC4784747; doi:10.1371/journal.pone.0151070)
Supplement: S6 Table — (DOCX) [file pone.0151070.s008.docx]

**Tables S6.**

| **Country** | **Subjects** | **N** | **Sampling year** | **AvDI_ENV_** | | | | |  | | **MEHP** | | | |  | | **MEOHP**  **(5oxo-)** | | | |  | | **MEHHP**  **(5OH-)** | | | | **Study** | |
| --- | --- | --- | --- | --- | --- | --- | --- | --- | --- | --- | --- | --- | --- | --- | --- | --- | --- | --- | --- | --- | --- | --- | --- | --- | --- | --- | --- | --- |
|  |  |  |  | **μg/kg_bw/day** | | **μg/day** | | |  | | **μg/g Cr** | | | |  | | **μg/g Cr** | | | |  | | **μg/g Cr** | | | |  | |
|  |  |  |  | **Mean** | | **Mean** | | |  | | **Mean** | | **50th** | |  | | **Mean** | | **50th** | |  | | **Mean** | | **50th** | |  | |
| Germany | 7-64 yrs | 85 | 2003 | | . | | . |  | | 12.4 | | . | |  | | 41.7 | | . | |  | | 57.2 | | . | | Koch et al., 2003 | |  |
| Germany | 3-14 yrs | 254 | 2001 | | . | | . |  | | 6.18 | | 5.85 | |  | | 31.2 | | 30.5 | |  | | 40.7 | | 39.9 | | Becker et al., 2004 | |  |
| Germany | 20-28 yrs | 60 | 2003 | | . | | . |  | | . | | 5.5 | |  | | . | | 12.0 | |  | | . | | 15.2 | | Wittassek et al., 2007 | |  |
| Denmark | Boys of 4-9 yrs | 503 | 2006-2007 | | . | | . |  | | 6.9. | | 6.8 | |  | | 27 | | 26 | |  | | 53 | | 52 | | Boas et al., 2010 | |  |
|  | Girls of 4-9 yrs | 342 | 2006-2007 | | . | | . |  | | 7.2 | | 6.7 | |  | | 29 | | 28 | |  | | 55 | | 52 | |  | |  |
| China | 21-49 yrs | 40 | 2011 | | . | | 182 |  | | . | | . | |  | | . | | . | |  | | . | | . | | Guo et al., 2011 | |  |
| India | 21-49 yrs | 22 | 2011 | | . | | 339 |  | | . | | . | |  | | . | | . | |  | | . | | . | |  | |  |
| Japan | 21-49 yrs | 35 | 2011 | | . | | 92.5 |  | | . | | . | |  | | . | | . | |  | | . | | . | |  | |  |
| Korea | 21-49 yrs | 60 | 2011 | | . | | 102 |  | | . | | . | |  | | . | | . | |  | | . | | . | |  | |  |
| Kuwait | 21-49 yrs | 46 | 2011 | | . | | 435 |  | | . | | . | |  | | . | | . | |  | | . | | . | |  | |  |
| Malaysia | 21-49 yrs | 29 | 2011 | | . | | 97.7 |  | | . | | . | |  | | . | | . | |  | | . | | . | |  | |  |
| Taiwan | Pregnant women (25-35yrs) | 99 | 2001-2002 | | . | | . |  | | 17.34 | | 16.37 | |  | | 36.97 | | 29.5 | |  | | 36.3 | | 33.2 | | Lin et al., 2011 | |  |
|  | 5 yrs | 59 | 2003-2004 | | . | | . |  | | 22.73 | | 21.38 | |  | | 63.9 | | 58.71 | |  | | 82.2 | | 69.05 | |  | |  |
|  | 2 yrs | 26 | 2006-2007 | | . | | . |  | | 27.5 | | 21.04 | |  | | 116.3 | | 114.33 | |  | | 140.4 | | 150.85 | |  | |  |
| Taiwan | Pregnant women (18-39 yrs) | 155 | 2000-2001 | |  | |  |  | | . | | 19.1 | |  | | . | | 25.6 | |  | | . | | 19.7 | | Lin et al., 2011 | |  |
| Taiwan | 3-9 yrs | 101 | 2007-2008 | | . | | . |  | | . | | 10.4 | |  | | . | | 44.0 | |  | | . | | 45.2 | | Hsu et al., 2012 | |  |
| Germany | 5-9 yrs | 104 | 2007-2009 | | . | | . |  | | 3.8 | | . | |  | | 25.6 | | . | |  | | 28.6 | | . | | Kasper-Sonnenberg  et al., 2012 | |  |
| Taiwan | 1-10 yrs | 29 | 2011, baseline | |  | |  |  | | . | | . | |  | | . | | 77.95 | |  | | . | | 84.7 | | Wu et al., 2013 | |  |
|  |  | 29 | 2011, 2M | |  | |  |  | | . | | . | |  | | . | | 43.92 | |  | | . | | 55.23 | |  | |  |
|  |  | 29 | 2011, 6M | |  | |  |  | | . | | . | |  | | . | | 23.4 | |  | | . | | 11.9 | |  | |  |
| USA | 6-11 yrs | 395 | 2011-2012 | | . | | . |  | | 2.02 | | 2.07 | |  | | 9.93 | | 9.89 | |  | | 14.9 | | 14.8 | | CDC, 2015 | |  |
|  | 12-19 yrs | 388 | 2011-2012 | | . | | . |  | | 1.53 | | 1.40 | |  | | 5.55 | | 5.21 | |  | | 8.33 | | 7.6 | |  | |  |
|  | >= 20 yrs | 1704 | 2011-2012 | | . | | . |  | | 1.51 | | 1.46 | |  | | 5.48 | | 5.19 | |  | | 8.61 | | 8.2 | |  | |  |
| Taiwan | Children | 227 | 2012 | | 8.2 | | 171.4 |  | | 13.1 | | 10.0 | |  | | 40.8 | | 32.2 | |  | | 58.4 | | 45.2 | | This study | |  |
|  | Adolescents | 12 | 2012 | | 3.2 | | 166.3 |  | | 6.3 | | 5.4 | |  | | 13.0 | | 10.3 | |  | | 19.0 | | 12.5 | |  | |  |
|  | Adults | 95 | 2012 | | 2.7 | | 174.5 |  | | 6.4 | | 3.5 | |  | | 10.2 | | 6.9 | |  | | 15.7 | | 10.2 | |  | |  |
|  |  |  |  | |  | |  |  | |  | |  | |  | |  | |  | |  | |  | |  | |  | |  |
