# Supplementary material for: Exposure Estimation for Risk Assessment of the Phthalate Incident in Taiwan
Source: PLoS One. 2016 Mar 9;11(3):e0151070. doi: 10.1371/journal.pone.0151070 (PMC4784747; doi:10.1371/journal.pone.0151070)
Supplement: S7 Table — (DOCX) [file pone.0151070.s009.docx]

**Table S7.**

|  |  | **Exposure group** | | |
| --- | --- | --- | --- | --- |
|  | **Low** | **Medium** | **High** | **Very High** |
|  | **(< 20 μg/kg_bw/day)** | **(20 - 50 μg/kg_bw/day)** | **(50 - 100 μg/kg_bw/day)** | **(>100 μg/kg_bw/day)** |
| Children | 0.54 (0.11-1.00) | 0.20 (0.05-0.86) | 0.11 (0.03-0.97) | 0.05 (0.01-0.41) |
| Adolescents | 0.52 (0.16-0.73) | 0.08 (0.08-0.09) | 0.06 | N/A |
| Adults | 0.32 (0.08-0.90) | 0.13 (0.03-0.94) | 0.03 (0.02-0.07) | 0.02 |
